# Effect of Reformation of the Anterior Chamber by Air or by a Balanced Salt Solution (BSS) on Corneal Endothelium after Phacoemulsification: A Comparative Study

**DOI:** 10.1155/2018/6390706

**Published:** 2018-04-08

**Authors:** Alahmady Hamad Alsmman, Mohammed Ezzeldawla, Amr Mounir, Ashraf Mostafa Elhawary, Osama Ali Mohammed, Mahmoud Farouk, Ahmed Mohamed Sherif

**Affiliations:** ^1^Department of Ophthalmology, Sohag Faculty of Medicine, Sohag University, Sohag, Egypt; ^2^Department of Ophthalmology, Cairo Faculty of Medicine, Cairo University, Cairo, Egypt

## Abstract

**Aim:**

To study the effect of reformation of the anterior chamber by air or by a balanced salt solution, after smooth phacoemulsification on the corneal endothelial count and morphology.

**Methods:**

A prospective interventional nonrandomized comparative study included 500 eyes of 500 patients with age range between 50 and 60 years, prepared for cataract surgery and presented to the Ophthalmology department of Sohag University Hospital in the period from October 2016 to May 2017. Corneal endothelial morphology and count were examined, and the results were recorded for all cases before the surgery. Patients were divided into two groups, and both groups were diagnosed with grade 2 cataract and underwent uncomplicated phacoemulsification performed by well-trained surgeons. At the end of the surgery, group 1 was subjected to a reformation of the anterior chamber via a balanced salt solution (BSS) injection while group 2 was subjected to a reformation of the anterior chamber via air injection. Corneal endothelial morphology and count were evaluated in the first and 3rd month postoperatively.

**Results:**

The study included 500 patients (250 in each group), 220 males (44%) and 280 females (56%) with no significant statistical age differences. Both preoperative and postoperative (3 months after the operation) recorded parameters of the corneal endothelium did not show any significant statistical differences. The cumulative dissipated energy was recorded, for all cases of both groups, during phacoemulsification with no significant statistical differences (*P* = 0.7).

**Conclusion:**

There is no difference between the effect of reformation of the anterior chamber after phacoemulsification, using air or using a BSS injection, on the corneal endothelial count and morphology.

## 1. Introduction

Cataract surgery is one of the most frequently performed surgeries worldwide. It is well established that this surgery has a negative effect on corneal endothelium, as it decreases the endothelial cell count. The severity of the affection depends on many variables, as phacoemulsification time and energy, surgical technique, anterior chamber depth, and the use of ophthalmic viscoelastic devices (OVDs) [1].

The corneal endothelium functions as an active pump and also as a barrier against the aqueous humor of the anterior chamber; thus, it holds an important role in the process of corneal tissue hydration. It has no ability of regeneration, so any decrease in its density is irreversible and can lead to permanent blurring of vision and pain [2].

The corneal injury caused by phacoemulsification usually leads to corneal edema, and if it is severe enough, it might result in irreversible bullous keratopathy, making corneal tissue transplantation the only effective treatment [2].

Corneal endothelial injury associated with phacoemulsification is assessed by specular microscopy through measuring changes of the cell density (CD) and the cell morphology [3].

Air injection has become widely used in many anterior segment surgeries [4], for example, restoration of normal intraocular pressure and reformation of the anterior chamber and many other surgical procedures [5].

Direct contact between gases and the corneal endothelial layer is not natural, and many experimental and clinical studies have proved the occurrence of corneal injury as a result of air injection into the anterior chamber [6].

Nowadays, most surgeons prefer to use a balanced salt solution to avoid the harms of air injection, even though there were no reported complications of air injection over the long term [7].

This study aims at comparing the effect of reformation of the anterior chamber after phacoemulsification, using air and BSS injection, on corneal endothelial count and morphology.

## 2. Patient and Methods

This is a prospective interventional nonrandomized comparative study, which included 500 eyes of 500 patients with age range between 50 and 60 years, prepared for cataract surgery and presented to the Ophthalmology department of Sohag University Hospital in the period from October 2016 to May 2017. The study only included cases diagnosed with grade 2 cataract according to the Lens Opacities Classification System III (LOCS III) [8].

Exclusion criteria included the following: patients aged less than 50 years or more than 60 years; patients with a history of previous corneal pathology, pseudoexfoliation, ocular trauma or intraocular surgery, or intraocular inflammation; and patients having preoperative endothelial cell count < 1500 cells per square millimeter, preoperative anterior chamber depth < 2.5 mm, or short axial length eyes < 21 mm and long axial length > 25 mm.

The ethical committee of Sohag University approved this study protocol, which was carried out according to the Declaration of Helsinki. A written informed consent was obtained from each included case.

All patients were divided into two groups; group 1 was subjected to phacoemulsification with a reformation of the anterior chamber using a balanced salt solution (BSS) injection at the end of the surgery, while group 2 was subjected to phacoemulsification with a reformation of the anterior chamber by air injection at the end of the surgery.

Patients who fit the criteria were allocated to each group in turn.

All patients were subjected to a full ophthalmological evaluation before the operation The evaluation included a slit-lamp examination, measurement of the best corrected visual acuity, measurement of the intraocular pressure (IOP), and specular microscopic examination (using Specular Microscope, SP-3000P, Topcon, Tokyo, Japan, with the IMAGEnet system (version 2.1, Topcon)). Also, central corneal endothelium morphology assessment was conducted, which includes central corneal thickness (CCT), endothelial cell density (ECD), corneal endothelial cell size variations as the percentage of the abnormal sizes (corneal polymegathism), and corneal cell shape variations as the percentage of the hexagonal cells (corneal pleomorphism). Postoperative follow-up occurred on the first day (after 6 hours), second day, one week, and one month and after three months.

Specular microscopic examination postoperatively occurred twice after one month and three months.

All patients received the same regimen of 1 drop of cyclopentolate 1%, 1 drop of phenylephrine 10%, and 1 drop of diclofenac 0.1% 20 minutes before surgery. Also, a dose of 5-6 ml lidocaine hydrochloride 2% with adrenaline 1 : 200,000 was used for the peribulbar anesthesia.

All operations were performed by three well-experienced surgeons (first three authors) using the standard divide and conquer technique and the same phacoemulsification equipment (INFINITI Vision System; Alcon Laboratories Inc., Fort Worth, TX, USA) at similar settings. Any cases that developed intraoperative complications were excluded from the study.

The patients were allocated to different surgeons in turn, the first surgeon 167 eyes, second surgeon 167 eyes, and third surgeon 166 eyes.

All cases were operated on using the same standardized surgical technique, which includes the use of a sterile drape with speculum, application of the corneal topical anesthesia (lidocaine 2% in gel suspension), performance of a 2.75 mm self-sealing corneal incision, injection of a viscoelastic agent (Healon; Advanced Medical Optics Inc., Santa Ana, CA), application of capsulorrhexis, application of hydrodissection, application of the conventional phacoemulsification (longitudinal ultrasound) (divide and conquer), irrigation and aspiration of the cortical material, introduction of viscoelastic in a bag to implant a foldable acrylic lens (AcrySof SA60AT; Alcon Laboratories), implantation of the lens through a 2.8 mm incision with the use of injector, and aspiration of the viscoelastic. Closure of the incision was done by hydration using a 30-gauge cannula with no sutures. The cumulative dissipated energy (CDE; phaco energy) was documented for all patients.

In the postoperative period, all patients received the same treatment including topical antibiotic (moxifloxacin) at a rate of 5 times per day for one week and prednisolone acetate at a rate of 5 times per day for one week with a gradual decrease in the second week, then replaced by nonsteroidal anti-inflammatory drops at a rate of three times per day for the following two weeks.

### 2.1. Statistical Analysis

The data were analyzed using SPSS for Windows version 18.0 software (SPSS Inc., Chicago, IL, USA). The data are shown as the mean and standard deviation. The results were analyzed using Student's *t*-test to compare the mean values of both groups. The qualitative data was expressed in the form of numbers and percentages and compared using the chi-square test. The multivariable regression analysis was done to identify the different corneal endothelial parameters. *P* value was considered significant if it was less than 0.05.

## 3. Results

The study included 500 patients (250 on each group), 220 males (44%) and 280 females (56%); group 1 included 120 males and 130 females with age range between 52 and 60 years, while group 2 included 100 males and 150 females with age range between 53 and 60 years. No significant statistical differences were recorded in the preoperative data about the age and the corneal parameters which include endothelial cell density, the coefficient of variance, hexagonality, and central corneal thickness (Table 1).

The cumulative dissipated energy was recorded for all cases during the phacoemulsification; the mean CDE in group 1 was 7.19 with SD 1.1, while the mean CDE in group 2 was 6.51 with SD 1. There was no significant statistical difference between both groups with *P* value = 0.7 (Table 1).

In group 1, the mean endothelial cell loss was 146 with SD 84 in the first month and 189 with SD 132 in the third month, while in group 2, the mean endothelial cell loss was 164 with SD 125 in the first month and 172 with SD 95 in the third month, with *P* value = 0.1 for both groups at the 3rd month.

**Figure 1:**
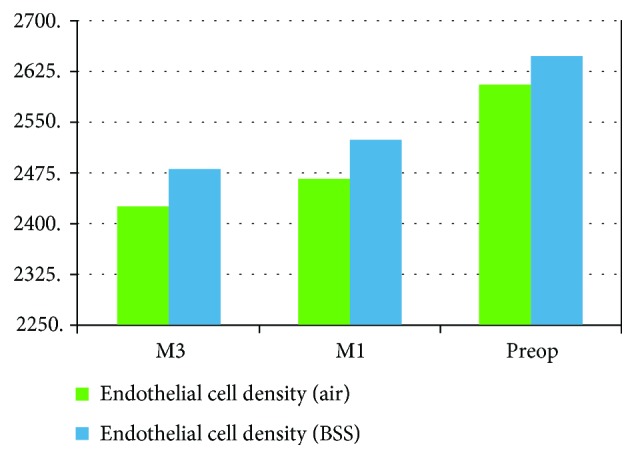
Pre- and post mean endothelial cell density.

**Figure 2:**
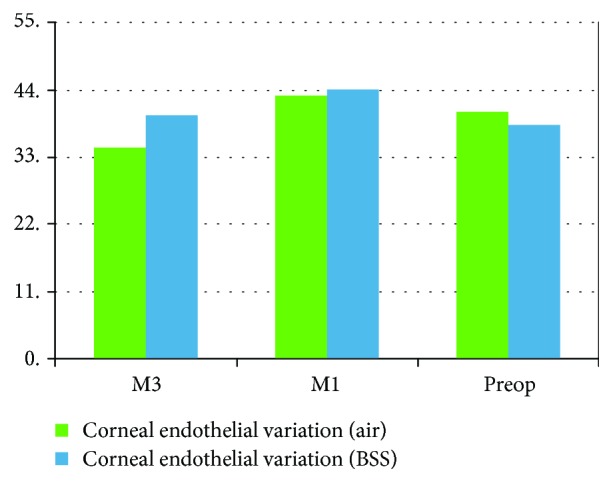
Pre- and post mean corneal endothelial variation.

**Figure 3:**
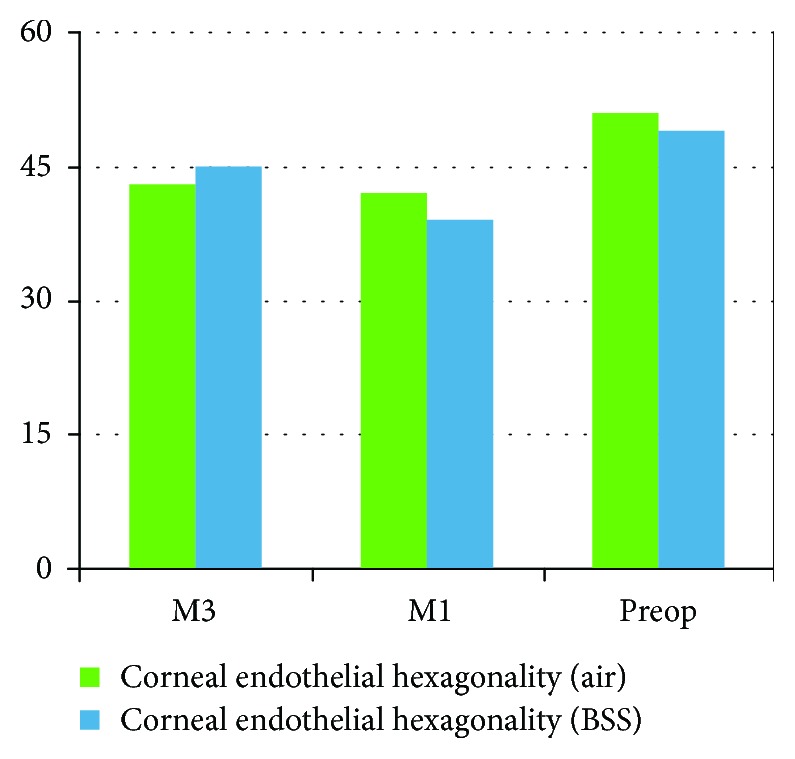
Pre- and post mean corneal endothelial hexagonality.

There were no operative complications reported for all cases. There was no significant statistical difference between both groups regarding the corneal endothelial data in the 3rd month postoperatively. The postoperative corneal parameters are summarized in Table 2 and Figures 1–3.

## 4. Discussion

Normally the cornea is transparent. This state is maintained by the corneal endothelium, which keeps the corneal stroma continuously dehydrated by acting as a barrier and an active fluid pump. This essential function is easily compromised by any damage that could happen during any eye surgery, especially phacoemulsification surgeries. This has prompted many studies to compare the severity of the damage that results from different cataract operation techniques [9].

Although the safety of the phacoemulsification has been markedly improved, the prevention of the corneal endothelial damage during phacoemulsification is still an important interest for all cataract surgeons [10].

In this study, we investigated the effect of reformation of the anterior chamber by air injection and by BSS injection on different corneal endothelial cell parameters, which were evaluated using the specular microscope to detect any damage resulting from the injection.

All preoperative data as regards age, the degree of cataract, and corneal endothelial cell parameters showed no statistical significance between both groups. Patients with other factors which might affect the corneal endothelial count were excluded, for example, patients older than 60 years and patients with advanced grades of cataract [11].

Sutureless corneal wounds have become the standard technique in cataract surgery, based on the fact that a watertight wound is an airtight wound and not vice versa. Hence, air injection is used for the reformation of the anterior chamber after phacoemulsification [12]. However, some studies reported air leakiness in 1/3 of the included cases [13].

In this study, we proved that there is no statistically significant difference between air and BSS injection in the anterior chamber reformation.

Our results agree with the study of Galin et al. [14] which were performed on rabbits' eyes. The authors examined the effect of air injection in the anterior chamber on the corneal endothelium. They used a light microscope and an electronic microscope for their study. They reported that the presence of air in the anterior chamber in contact with the corneal endothelium has no toxic effect on the corneal endothelium but even stimulates the proliferation of the corneal endothelial cells.

Also, our results are similar to the results of the Ventura et al. study [15], who confirmed that the air has no damaging effect on the corneal endothelium of the cat. Our results coincide with the results of the Norn study [5]. Norn studied the effect of reformation of the anterior chamber, after cataract extraction, using air injection on the corneal endothelium of humans. His study included an examination of the patients before and after the surgery. He proved that the corneal thickness was thinner in patients injected with air with no other adverse effects over a six-month period following the operation.

This study disagreed with the study of Olson et al. [16], who compared the effect of air and balanced saline solution injection into the anterior chamber on the corneal endothelium of cats. They reported a significant decrease in the endothelial cell density after air injection into the anterior chamber. He also noted significant endothelial damage during corneal perfusion studies.

Corneal pachymetry is assessed as the occurrence of edema is an indirect tool to evaluate corneal endothelial changes. It is important in cases of surgically induced endothelial cell loss [17].

Although, in this study, an initial increase in the corneal thickness caused by postoperative edema was reported. But the difference between both groups was not of statistically significance.

A significant positive correlation was found in many studies between the endothelial cell loss and the nuclear sclerosis grade, also between the endothelial cell loss and phacoemulsification power and time [18]. So only cases with grade 2 cataract were included in this study. Also, the power used in the surgery did not show a significant difference between both groups. So the endothelial cell loss reported in our study was not affected by the previous factors, and as a result, the effect of air on the corneal endothelium had not been masked by any factor.

Corneal endothelial parameters as regards cell density, endothelial cell loss, hexagonality, and coefficient of variance did not show any significant difference between both groups, which means that the reformation of the anterior chamber by air injection has no toxic effect on the corneal endothelium. This was expected as thousands of phacoemulsification surgeries with air use in the anterior chamber reformation have been performed in our society “South Egypt” with satisfactory results.

## 5. Conclusions

There is no difference between the effect of reformation of the anterior chamber, after phacoemulsification, using air or using BSS injection on the corneal endothelial count and morphology. Also, there is no reported toxic effect of air on corneal endothelial parameters evaluated by specular microscope.

## Figures and Tables

**Table 1:** Preoperative demographics and corneal endothelial parameters.

	Group 1 (BSS)		Group 2 (air)		
	Mean ± SD	Range	Mean ± SD	Range	*P* value
Sample size (male : female)	250 (120 : 130)		250 (100 : 150)		
Age (year)	56 ± 2.3	52 to 60	57.9 ± 3.9	53 to 60	0.1
CDE (joules)	7.19 ± 1.1	4.96 to 9.14	6.51 ± 1	4.97 to 8.52	0.7
Cell density (cells/mm^2^)	2646 ± 284	1858 to 2890	2604 ± 367	1998 to 3209	0.16
Coefficient of variance % (polymegathism)	38.2 ± 6	27 to 49	40.3 ± 8.4	31 to 61	0.8
Hexagonality % (pleomorphism)	49 ± 8	39 to 64	51 ± 10	34 to 73	0.3
Central corneal thickness (micron)	508 ± 22	474 to 571	509 ± 34	450 to 571	0.28

**Table 2:** Pre- and postoperative corneal endothelial parameters.

	Group 1 (BSS) Mean ± SD			Group 2 (Air) Mean ± SD			*P* value
	Preoperative	Post 1 month	Post 3 month	Preoperative	Post 1 month	Post 3 month	
Cell density (cells/mm^2^)	2646 ± 284.1	2523 ±311	2479 ± 303	2605 ± 367	2465 ± 351	2424 ± 336	0.16
Endothelial cell loss	—	146 ± 84 5.5%	189 ± 132 7.1%	—	164 ± 125 6.3%	172 ± 95 6.6%	0.1
Coefficient of variance % (polymegathism)	38.2 ± 6	1644±	39.8 ± 10	40.3 ± 8.4	35.8 ± 11	34.4 ± 11	0.8
Hexagonality % (pleomorphism)	49 ± 8	39 ± 15	45 ± 10	51 ± 10	42 ± 8	43 ± 9	0.3
Central corneal thickness, micron	508 ± 22	518 ± 23	508 ± 20	509 ± 34	530 ± 40	510 ± 41	0.28
